# Electrochemical Study of Biotin-Modified Self-Assembled Monolayers: Recommendations for Robust Preparation

**DOI:** 10.1100/tsw.2006.20

**Published:** 2006-01-17

**Authors:** Richard J.C. Brown, Dan J.L. Brett

**Affiliations:** ^2^ Department of Chemical Engineering and Chemical Technology, Imperial College, London, SW7 2AY, USA; ^1^ Analytical Science Group, National Physical Laboratory, Teddington, Middlesex, TW11 0LW, UK

**Keywords:** self-assembly monolayers, biotin functionalisation, impedance spectroscopy

## Abstract

The development of the underpinning methodology for the production of robust, well-formed, and densely packed biotin-HPDP functionalised gold surfaces, the crucial first step in immobilising bimolecules on surfaces, is described. Self-assembled monolayers (SAMs) with biotin end-groups were prepared on polycrystalline gold surfaces according to a published method. The layers formed were studied using cyclic voltammetry to determine the composition of the layer and its quality. Crystal impedance spectroscopy was also applied as a complimentary indicator of the composition of the layer.For the first time, the effect of assembly time on the properties of the layer was studied along with the composition of the layer and the ability of the precursor molecule to self-assemble by oxidative addition.

## INTRODUCTION

The underlying motivation for this work was to develop a robust methodology to functionalise gold surfaces with Biotin derivatives with a view to using such surfaces for SPR and TIRF applications. (Biotinylation is the accepted first step in the modification and functionalisation of surfaces for biological compatibility[1].) Development of this understanding required a fundamental investigation of the underlying physical and chemical parameters, which affected the quality, stability, and effectiveness of surface functionalised in this manner. Several electrochemical techniques were used in an attempt to elucidate this system.

Zimmermann and Cox[2] previously developed a method to functionalise bare gold surfaces with modified Biotin self-assembled monolayers (SAMs). The same methodology was employed to create test surfaces for the investigations reported here.

This method involves the reduction of the disulphide bridge in Biotin-HPDP[3] (N-[6-(Biotinamido)hexyl]-3'-(2'-pyridyldithio) propionamide) (see Fig. 1) by tri-butylphosphine to yield a biotin species with an alkane thiol functionality, and 2-thio-pyridine, in solution.

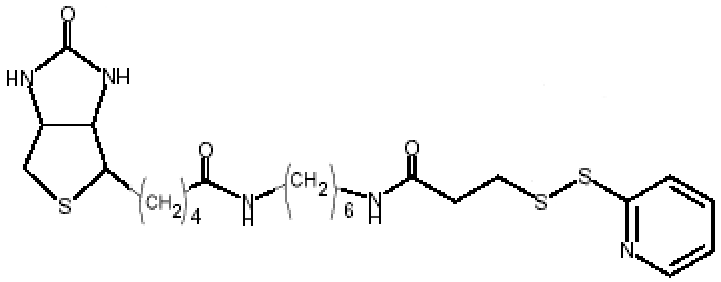

**FIGURE 1**. Biotin-HPDP. The cleavable disulphide bridge is at the right-hand side of the molecule, near to the pyridinyl ring.

A bare gold substrate was then immersed in the above solution yielded by the above procedure to perform the surface modification. The alkane thiol functionality on the modified biotin should form a SAM on the gold surface. The 2-thio-pyridine, also in solution, may also self-assemble on the gold surface, but the extent to which this occurs is not known, and whether this process interferes with the production of a robust biotin-modified SAM. In order to elucidate this, the electrochemical properties of the biotin-modified SAM, and the time dependency of these properties, were compared against a variety of well-characterised "control" or "calibration" SAMs.

The electrochemical properties of the SAMs created from the Biotin-HPDP using the preparative procedure outlined above were compared to the electrochemical properties of SAMs prepared using alkane thiols with carbon chain lengths of C6, C12, and C18. These SAM layers have extremely well-known preparations and physicochemical characteristics[4].

The electrochemical properties of SAMs prepared using benzene thiol were also investigated. Benzene thiol was used as an appropriate compound to replicate the self-assembly properties of 2-thio-pyridine, which is not commercially available.

## EXPERIMENTAL

SAMs with biotin end-groups were prepared on polycrystalline gold surfaces according to the method described above[2]. These layers were studied using cyclic voltammetry to determine the composition of the layer and its quality. Crystal impedance spectroscopy was also used as a complimentary indicator of the composition of the layer and also because it is of interest as a possible transduction method for sensing the interaction between the functionalised surface and binding analyte species as a complementary technique to Surface Plasmon Resonance (SPR).

The effect of assembly time on the properties of the layer was studied along with the composition of the layer and the ability of the precursor molecule to self-assemble by oxidative addition. For voltammetric experiments, various assembly times for the self-assembly process for biotin-HPDP were studied, whereas 1 h was used consistently for the crystal impedance measurements. Assembly of benzene thiol and "calibration" thiol layers, produced with the C6, C12, and C18 alkane thiols, were performed from 1 m*M* of thiol in ethanol and the assembly time was in excess of 2 h.

The quality of the functionalised layers was assessed by electrochemical reductive desorption in alkaline electrolyte. For these investigations, the electrode substrate used was a polycrystalline gold disc with a diameter of 7 mm. The voltammetry performed employed a conventional three-electrode cell with platinum counter electrode and Saturated Calomel Electrode (SCE) as reference. The reference electrode was isolated from the main cell using a Luggin-Haber capillary so as to avoid contamination of chloride anions from the reference. The electrode was prepared by mechanical polishing using a slurry of alumina powder (0.3 μm) to achieve a mirrored finish. Remaining alumina was removed by sonication and the electrode was "electrochemically polished" by cycling in 0.2 *M* sulphuric acid solution between –0.2 and

1.6 V vs. SCE at 50 $mVs^{-1}$ until a voltammetry profile was reached that is characteristic of a clean electrode.

The true surface area of the electrode was determined by reversing the sweep of the cyclic voltammetry response while electrochemically polishing the electrode at the Burshtein minimum (ca. 1.42 V vs. SCE) and using the oxide reduction peak to calculate the surface area from a knowledge of the charge per unit area for the reduction of oxide on polycrystalline gold. The degree to which surface roughness increases the surface area of an electrode is described by the Roughness Factor (RF) and is the ratio between the true "rough" surface of the electrode and the geometric surface area. The roughness factor of the electrode used for the voltammetry was calculated to be 2.30 ± 0.25.

For the impedance measurements, all crystals used were nominal 10 MHz AT-cut and supplied by International Crystal Manufacturing Company Inc., Oklahoma City. The crystals are calibrated to ±2% of the fundamental frequency and have dimensions as shown in Fig. 2. The quartz used as the gold substrate in this study were of the Cr underlayer type; the gold used was 99.99% pure and vacuum deposited to form a 100-nm thick layer. Connecting wires are spring bonded to the flag of each electrode.

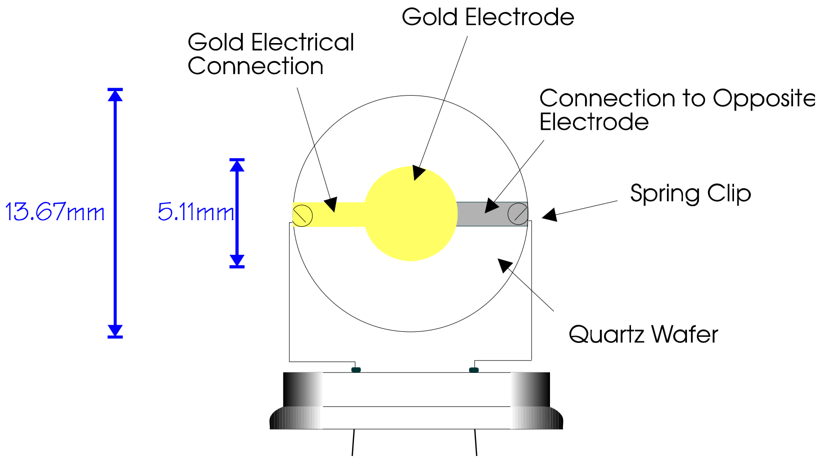

**FIGURE 2**. Dimensions of the EQCM crystal.

Crystal impedance measurements were performed using a frequency response analyser (Solartron 1260 with ZPlot software). An initial frequency scan was performed between $9.975 \times 10^6$ and $1.0025 \times 10^7$ Hz to obtain the frequency region of the fundamental oscillation. A subsequent scan was then performed close to the fundamental oscillation frequency with a higher number of frequency points to obtain an accurate characterisation of the admittance close to oscillation. Fig. 3 shows an example of the admittance (inverse impedance) characteristic of a clean, bare electrode and the same electrode after self-assembly of a thiol with a carbon chain length of 18 ($C_{18}$). Both the wide frequency scan and the accurate narrow frequency scan are shown. The characteristic frequency was taken as that at the maximum conductance; this is the *true* resonance frequency of the system as opposed to the zero phase frequency used in conventional QCM detectors.

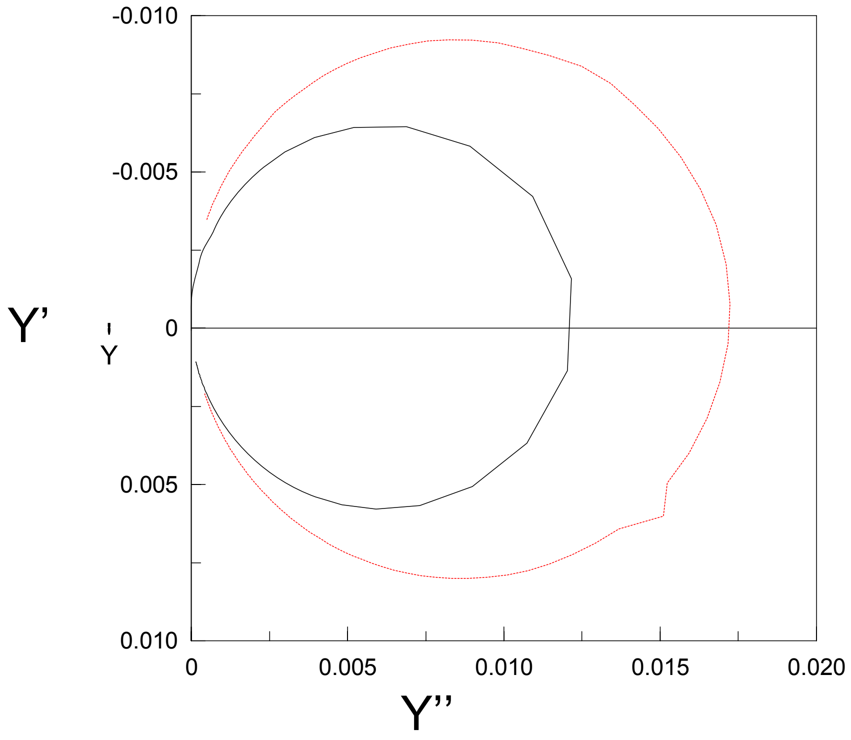

**FIGURE 3(a).** Complex plane admittance plot of $C_{18}$ (dashed red line) and bare gold (solid black line) at low resolution between $9.975 \times 10^6$ and $1.0025 \times 10^7$ Hz.

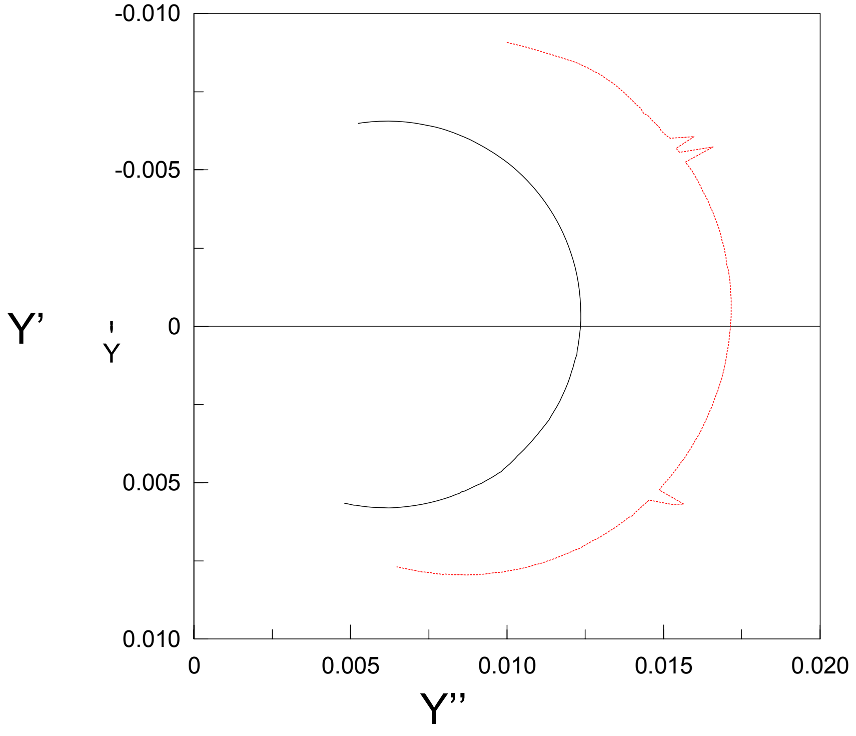

**FIGURE 3(b).** Complex plane admittance plot of $C_{18}$ (dashed red line) and bare gold (solid black line), at high resolution, close to the fundamental oscillation.

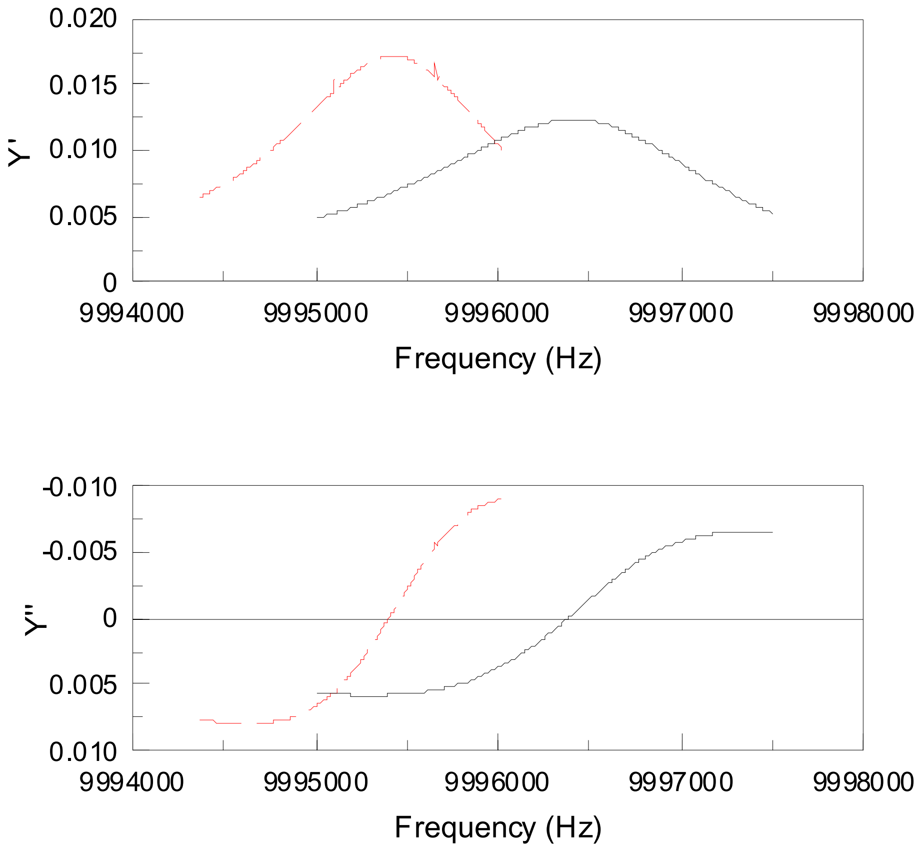

**FIGURE 3(c).** Bode plots of $C_{18}$ (dashed red line) and bare gold (solid black line) close to the fundamental oscillation.

## RESULTS AND DISCUSSION

### Crystal Impedance Spectroscopy

The frequency shifts (mass loading/film thickness) caused by surface functionalisation using three different alkane thiols with carbon chain length of 6, 12, and 18 ($C_6$, $C_{12}$, and $C_{18}$) were measured to provide a reference against which the layer formed from the biotin-HPDP precursor could be compared. The response for a benzene thiol was also studied, since this is expected to be similar to that of a pyridine thiol that may form as part of a mixed monolayer system, during the chemical reduction with tri-n-butyl phosphine. Fig. 4 shows the frequency shift (of the point of maximum conductance) that results from surface functionalisation using each of the "calibration" alkane thiols. This plot is a calibration relationship for ideal surface functionalisation using "straight chain" thiols. Equivalent Alkane Thiol Chain Lengths (EATCL) have then been determined for surface functionalisation using benzene thiol and biotin-HPDP by plotting the experimentally observed frequency shift for these thiols onto the calibration curve.

It can be seen that the response for the $C_6$, $C_{12}$, and $C_{18}$ alkane thiols fits a quadratic curve. It would be expected that chain length and frequency shift should scale linearly since the crystal impedance measurement is sensitive to the thickness of the layer. However, it is known that longer layers form denser, better formed layers, (for a given assembly time) due to the greater interchain interactions between the adjacent alkane chains. Therefore, larger frequency shifts may be expected to occur for longer chain thiols and lead to the positive deviation in the curve observed in Fig. 4.

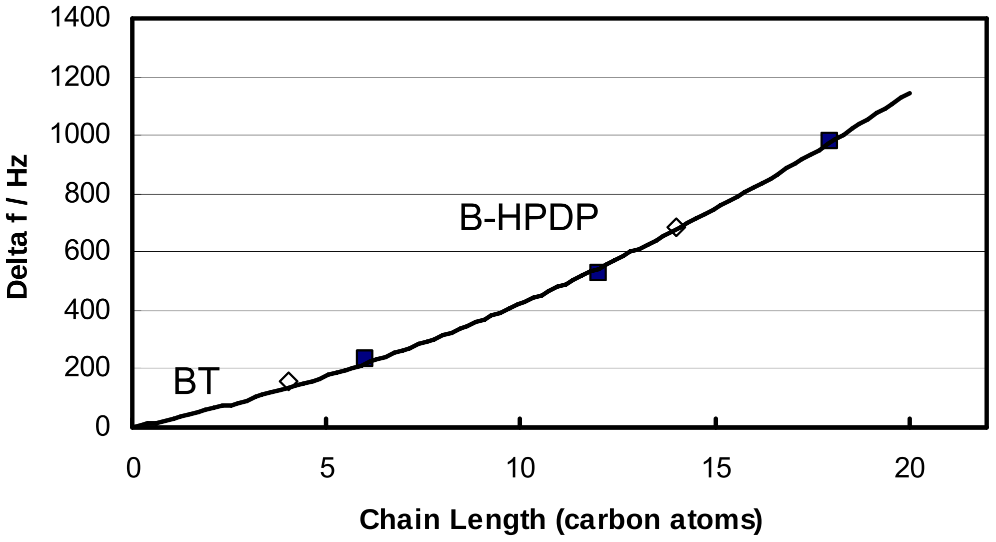

**FIGURE 4.** Frequency shift in the maximum conductance of the admittance against carbon chain length for "calibration" alkane thiols (■) of $C_6$, $C_{12}$, and $C_{18}$. EATCLs for benzene thiol (◊ BT)– and biotin-HPDP (◊ B-HPDP)–derived SAMs have then been interpolated from experimentally observed frequency shifts.

When the frequency shift for the benzene thiol– and the biotin-HPDP–derived layers are plotted onto the calibration curve, it can be seen that the benzene thiol corresponds to an EATCL of approximately 4. Considering the size of the aromatic group, this is within the range of what would be expected, and implies that the layer produced from benzene thiol is formed in an analogous fashion to those of alkane thiols, i.e., the benzene group does not form flat on the surface, but is close packed in a direction approximately normal to the surface of the electrode.

The biotin-HPDP–derived layer forms with an EATCL close to 12. This is significantly shorter than would be expected to form based on the free molecule size (which would indicate an expected EATCL of 21+). This may be due to several reasons. Firstly, it is possible that the layer forms as a mixed monolayer of pyridine thiol– and biotin-derivatised thiol as both species are produced during chemical reduction of the biotin-HPDP. This would effectively reduce the thickness of the layer, as sensed by the crystal impedance technique. Alternatively, the biotin thiol may not self-assemble in an orderly way, analogous to that of straight-chain thiols. This may result in the layer being less dense and effectively thinner than would be expected based on the length of the free molecule. This may occur because the kinetics of the self-assembly process are slower than that of a simple alkane thiol, and consequently 1 h is not long enough for a dense, well-packed SAM to form. The presence of the two amide groups along the chain is also likely to decrease the ability of the chains to close pack and will interfere with the interchain dispersive forces. Additionally, the bulky end-group of the biotin moiety may preclude well-packed SAM formation on steric grounds. Poor SAM formation due to bulky head-groups has been reported previously for ferrocene-derived thiols[4].

## Voltammetry of SAMs (Reductive Desorption)

Reductive desorption of the biotin-HPDP–derived layers was performed to assess the quality of the SAM, the amount of thiol on the surface, and to determine if the layer forms as a single component system. The effect of assembly time was also investigated.

To establish if the biotin-HPDP–derived layer forms as a single component system, the voltammetry of a benzene thiol–derived SAM was investigated to give an impression of the kind of response that would be expected if pyridine-2-thiol coassembled. Fig. 5 shows the reductive desorption response of a benzene-thiol SAM taken in 0.1 *M* NaOH at a scan rate of 20 $mVs^{-1}$. The reduction peak at ca. -1.00 V is that of the thiol being removed from the surface. The smaller oxidative peak at ca. –0.90 V is due to some of the thiol going back down onto the surface.

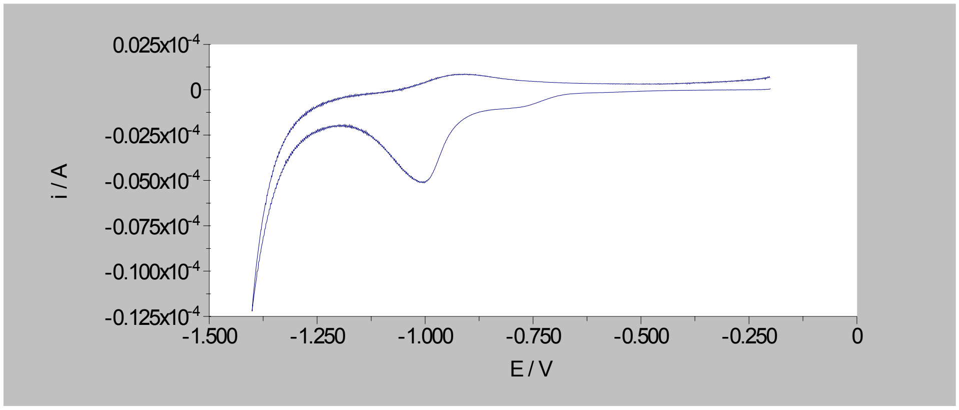

**FIGURE 5.** Reductive desorption of benzene thiol in 0.1 *M* NaOH at a scan rate of 20 $mVs^{-1}$.

The reduction wave (or prewave) at ca. –0.78 V is thought to be due to the incorporation of electrolyte into the layer prior to reductive desorption (i.e., a nonfaradaic process). However, it may be possible that benzene thiol exhibits unusual behaviour (compared to long-chain thiols) and reductively desorbs over a broad range of potentials or takes part in a structural reorganisation (phase transition). For long-chain thiols, the prewave is observed more markedly for poorly formed layers, and is more likely to occur for shorter-chain SAMs (i.e., for a system with an EATCL of approximately 4).

The reductive desorption characteristics of the SAMs derived from biotin-HPDP after assembly times of 0.5, 60, and 1440 min was determined. The electrochemical reductive depsortion for assembly times of 60 and 1440 min are shown in Fig. 6. It is observed that the reductive desorption peaks (at –1.200, –1.226, and –1.250 V for 0.5, 60, and 1440 min, respectively) become more negative with increasing immersion time. It is not possible to integrate all of the reductive desorption peaks accurately due to the sloping baseline caused by exposure of bare electrode to the electrolyte during thiol removal (with a corresponding increase in double-layer capacitance) and overlay of the solvent reduction peak. However, taking the peak currents, they are seen to increase in size with increasing immersion time ($0.341 \times 10^{-5}$, $0.772 \times 10^{-5}$, and $0.122 \times 10^{-4}$ A for 0.5, 60, and 1440 min, respectively). The increase in the reduction current and the shift to more negative reduction potentials shows that the SAM continues to improve in quality up to 24 h after initial immersion. This is significantly longer than the suggested self-assembly time reported by Zimmermann and Cox[2].

Increasing the time allowed for self-assembly shows that the size of the reductive desorption prewave decreases in size. The prewave is a measure of how intact the layer is in terms of the ability of electrolyte to be incorporated into the layer. However, for the system studied here, the possibility exists that a mixed monolayer may form, composed of the biotin-HPDP thiol and a pyridine-2-thiol. In which case the "prewave" will have a component due to the reductive desorption of the pyridine-2-thiol. It is seen in Figs. 6(a) and 6(b) that a small peak is discernable at ca. –1 V, at a potential close to that of the reductive desorption of benzene thiol shown in Fig. 5. This is attributed to the reductive desorption of pyridine-2-thiol. According to the voltammetric response, the pyridine-2-thiol only constitutes a small portion of the layer and becomes negligible for the longest adsorption time. This is expected since shorter EATCL species will tend to be displaced by longer-chain thiols, which have a greater interchain interaction and therefore form lower energy layers.

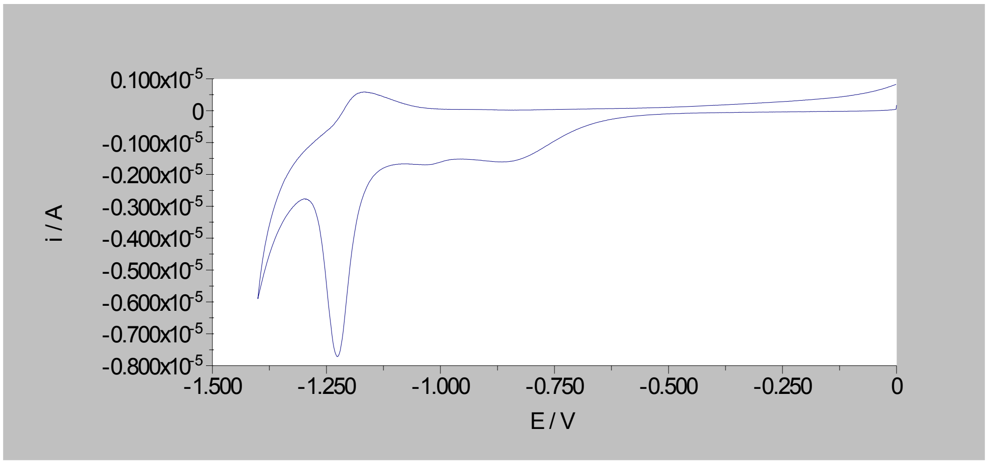

**FIGURE 6(a).** Reductive desorption of biotin-HPDP in 0.1 *M* NaOH at a scan rate of 20 $mVs^{-1}$. Assembly time of 60 min.

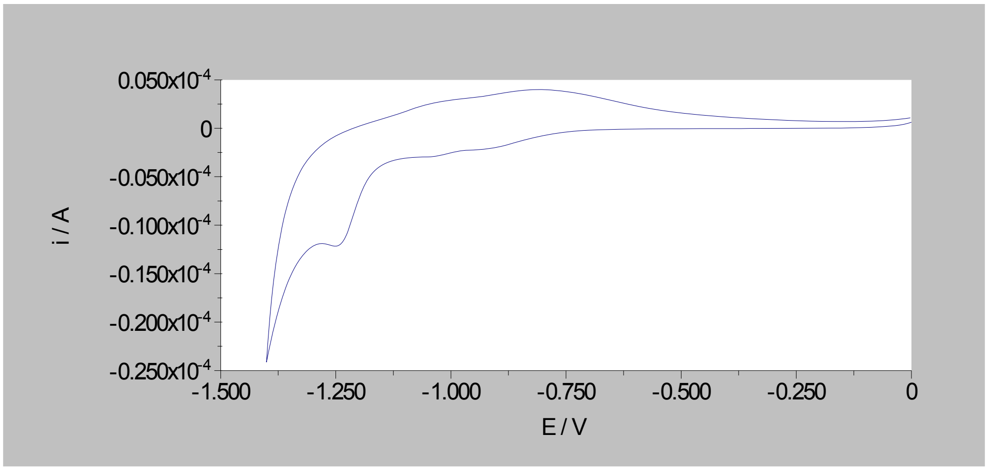

**FIGURE 6(b).** Reductive desorption of biotin-HPDP in 0.1 *M* NaOH at a scan rate of 20 $mVs^{-1}$. Assembly time of 1440 min.

There are conflicting reports as to the form in which the pyridine half of the disulphide part of the biotin-HPDP exists subsequent to chemical reduction. Zimmerman and Cox[2] claim that a pyridine-2-thiol forms that is expected to react at the surface of the gold to form a mixed monolayer with the biotin-HP-thiol. While the EZ-Link™ biotin-HPDP application sheet and work by Butt et al.[5] claim that a pyridine-2-thione forms that is not expected to be as active as the thiol to self-assembly, although rearrangement may occur to form the thiol. The possibility of forming a mixed monolayer with the product of the pyridine part of the biotin-HPDP after chemical reduction can be removed by purification of the biotin-HPDP thiol using gel filtration or diafiltration[5].

It should be noted that despite the biotin-HPDP-thiol SAM forming an effectively monocomponent layer after self-assembly time in the order of 24 h, the prereduction wave shows that the layer is not well formed compared to that of an alkane thiol of similar length. The layer exhibits voltammetry indicative of a poorly formed SAM that allows electrolyte incorporation into the layer prior to reductive desorption. The crystal impedance data also imply that the biotin-HPDP–derived SAM forms in an inferior way to long-chain alkane-thiol SAMs. The use of a mixed monolayer system is therefore recommended to facilitate close packing and proper presentation of the biotin moiety to the analyte, i.e., a mixed monolayer will leave the biotin group less crowded and more exposed at the surface.

To investigate the possibility of using the biotin-HPDP directly and without chemical reduction with tri-n-butyl phosphine, self-assembly of the biotin-HPDP was performed from ethanolic solution. A self-assembly time of 1 h resulted in the reductive desorption voltammetry shown in Fig. 7.

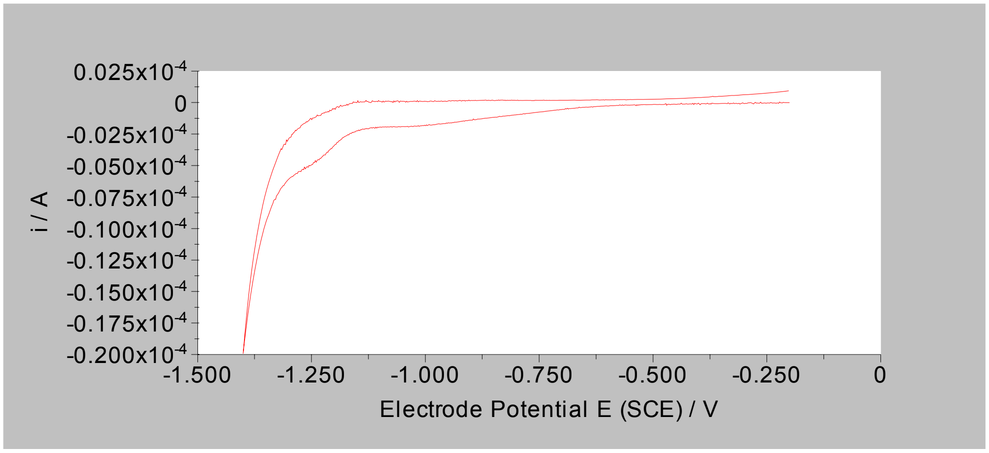

**FIGURE 7**. Reductive desorption of biotin-HPDP, formed by direct assembly without chemical reduction, in 0.1 *M* NaOH at a scan rate of 20 $mVs^{-1}$. Assembly time of 60 min.

Disulphides are known to be able to react directly with gold via oxidative addition[4]. However, as can be seen from the small size of the reductive desorption peak, self-assembly does not occur as fast as via the chemical reduction to thiol route. This is most likely due to the steric hindrance caused by having the long-chain biotin on one side of the disulphide and pyridine on the other. To react, both of the sulphur atoms must come into close proximity to the gold surface. This will become progressively less likely as the layer forms.

It is concluded , therefore, that oxidative addition is not a viable route to the formation of well-packed layers for the biotin-HPDP precursor due to the slower kinetics and greater chance of formation of a mixed monolayer (due to the pyridine moiety not being converted to pyridine-2-thione by the chemical reduction).

## CONCLUSIONS

This experimental investigation has led to several conclusions on the nature of biotin-HPDP functionalisation at gold surfaces and proposes several best-practice solutions to ensure the production of robust, well-formed, and densely packed biotin-HPDP functionalised gold surfaces based on conclusions from experimental evidence:

- The robust “electrochemical polishing” procedure, developed to ensure that gold surfaces are clean before functionalisation, has been shown to be successful.
- The biotin-HPDP forms a poorly packed SAM on the gold surface, in comparison to straight-chain alkane thiols, with an EATCL of about 12.
- This poor monolayer packing is caused by the chemical composition of the biotin-HPDP molecule, which is less amenable to the formation of stable monolayers than straight-chain thiols. It is also caused by the initial presence of a mixed monolayer incorporating pyridinyl thiol, as a by-product of the initial chemical reduction.
- The quality of the layer continues to improve for a considerable period after initial deposition.

- The biotin-HPDP functionalised surfaces should be allowed a minimum of 24 h to form before further experimental use.
- Reductive desorption experiments show that layer formation by oxidative addition, without initial chemical reduction, is not suitable for functionalisation.
- Electrochemical crystal impedance spectroscopy shows excellent promise as a transduction method for sensing the interaction between the functionalised surface and binding analyte species.

## ACKNOWLEDGEMENTS

This work was funded by the U.K. DTI National Measurement System's Valid Analytical Measurement Programme.

## BIOSKETCH

**Dr. Richard J.C. Brown** is a Principal Research Scientist in the Analytical Science Group at the National Physical Laboratory in the U.K. Dr. Brown was born in Epsom, U.K. in 1975 and was educated at Epsom College and Imperial College, London, from where he received his BSc in Chemistry (1st Class Hons.) together with the Governors' Prize for Chemistry in 1997. He remained at Imperial, where he was awarded his PhD in Electrochemistry in 2000 together with the Physical Chemistry Research Prize. He then moved to the National Physical Laboratory in Teddington, U.K., as a Higher Research Scientist, where he assumed responsibility for maintaining the U.K.'s pH measurement standards, developing ultra-low reflectance surfaces (later to receive widespread media and scientific interest as "NPL Super Black"), and developing methodologies for accurate air quality measurement. His work on NPL Super Black earned him NPL's Silver Medal in 2001 and NPL's Rayleigh Award in 2002. Dr. Brown's work on the accurate measurement of trace metals in ambient air earned him NPL's Silver Medal in 2005.

His interests include the measurement of heavy metals in ambient air at ultra-low concentrations, the use of surface-enhanced Raman spectroscopy as a quantitative trace analysis technique, pH measurement standards, and the uncertainty analysis of pH measurements made using the Harned cell. He also represents the U.K. on the international Electrochemical Analysis Working Group of the BIPM's CCQM, and on three European standardisation committees for the measurement of organic and metallic pollutants in ambient air. Dr. Brown is a Member of the Royal Society of Chemistry, a Chartered Chemist, and an Academic Visitor in the Faculty of Physical Sciences at Imperial College.
